# A survey of tobacco dependence treatment guidelines in 121 countries

**DOI:** 10.1111/add.12158

**Published:** 2013-04-22

**Authors:** Hembadoon Piné-Abata, Ann McNeill, Martin Raw, Asaf Bitton, Nancy Rigotti, Rachael Murray

**Affiliations:** 1 UK Centre for Tobacco Control Studies, University of Nottingham, Division of Epidemiology and Public HealthNottingham, UK; 2 UK Centre for Tobacco Control Studies, National Addiction Centre, Institute of Psychiatry, King's College London, Addiction Sciences BuildingLondon, UK; 3 Division of General Medicine, Brigham and Women's Hospital, Department of Health Care Policy, Harvard Medical SchoolBoston, MA, USA; 4 Harvard Medical SchoolBoston, MA, USA; 5 Tobacco Research and Treatment Centre, General Medicine Division, Massachusetts General HospitalBoston, MA, USA

**Keywords:** Article 14, Article 14 guidelines, FCTC, national treatment guidelines, survey, tobacco dependence treatment

## Abstract

**Aims:**

To report progress among Parties to the World Health Organization (WHO) Framework Convention on Tobacco Control (FCTC) in developing national tobacco treatment guidelines in accordance with FCTC Article 14 guideline recommendations.

**Design:**

Cross-sectional study.

**Setting:**

Electronic survey from December 2011 to August 2012; participants were asked to complete either an online or attached Microsoft Word questionnaire.

**Participants:**

One hundred and sixty-three of the 173 Parties to the FCTC at the time of our survey.

**Measurements:**

The 51-item questionnaire contained 30 items specifically on guidelines. Questions covered the areas of guidelines writing process, content, key recommendations and other characteristics.

**Findings:**

One hundred and twenty-one countries (73%) responded. Fifty-three countries (44%) had guidelines, ranging from 75% among high-income countries to 11% among low-income countries. Nearly all guidelines recommended brief advice (93%), intensive specialist support (93%) and medications (96%), while 66% recommended quitlines. Fifty-seven percent had a dissemination strategy, 76% stated funding source and 68% had professional endorsement.

**Conclusion:**

Fewer than half of the Parties to the WHO FCTC have developed national tobacco treatment guidelines, but, where guidelines exist, they broadly follow FCTC Article 14 guideline recommendations.

## Introduction

Article 14 of the World Health Organization (WHO) Framework Convention on Tobacco Control (FCTC) ‘Demand reduction measures concerning tobacco dependence and cessation’, requires Parties to develop and disseminate comprehensive guidelines based on scientific evidence, and to take effective measures to promote cessation of tobacco use and adequate treatment for tobacco dependence 1. In November 2010, the fourth conference of the Parties to the FCTC adopted guidelines for the implementation of Article 14. These guidelines amount to official policy on tobacco dependence treatment for Parties to the Convention, and, inter alia, outline the key characteristics of national guidelines, which should 1:

1. Be comprehensive—include a broad range of interventions, such as the identification of tobacco users and the provision of brief advice, behavioural support provided by telephone quitlines and face-to-face, access to free or affordable medications, and cover all settings and all providers within and outside the health-care sector
2. Be evidence based
3. Include a dissemination and implementation plan
4. Emphasise the importance of all service providers setting a good example by not using tobacco and that they should be helped to stop
5. Be developed through active collaboration between government and other relevant stakeholders, including health professional organisations, and be endorsed widely at national level, including by health professional organisations
6. Be protected from conflicts of interest during their development
7. Be reviewed periodically and updated as necessary.

In this article we report the results of a survey whose objective was to review the state of tobacco dependence treatment systems and treatment guidelines among Parties to the FCTC in order to gauge progress in implementing Article 14 and the Article 14 guidelines. The survey builds on a previous survey whose results were published in 2009 2,3. We report here the guidelines results; the treatment systems results are reported in a separate article (submitted for publication).

## Methods

### Sample

The 2009 study 2 used a convenience sample of 31 countries. For this study we sought to survey all Parties to the FCTC. We developed a contact list in a stepwise way, starting with contacts from the last survey and other contacts in the treatment field known to us, including through the FCTC Article 14 working group. We then used contacts from the Framework Convention Alliance (FCA), a civil society alliance that promotes FCTC implementation, and Global Bridges, a network of health-care professionals that advocate effective tobacco control policies and run training courses. Finally, we found some contacts with help from WHO regional offices. Our contacts were a mixture of treatment specialists, FCA members and government officials.

At the time of our survey there were 174 Parties to the FCTC. This number included the European Union (EU), as well as its 27 member states, so we excluded the EU (which obviously cannot have national guidelines), leaving 173 Parties. From the 173 Parties we were unable to find contacts in 10 Parties, thus 163 Parties were surveyed. The UK, which is a Party, consists of four countries—England, Northern Ireland, Scotland and Wales—each with separate health-care systems and treatment guidelines, so we surveyed all four individually. Our final sample therefore consisted of 163 FCTC Parties or 166 countries.

We emailed 166 people, starting in December 2011, inviting them to participate in our survey by clicking on a link to the online survey contained in the email, or by completing an attached Microsoft Word questionnaire (offered in English, French and Spanish). We followed up non-responders with reminder emails in January, February, April and May 2012.

Where we needed clarification of answers we corresponded with contacts, until mid-August, when the survey was closed.

Countries were categorised by WHO region, and World Bank income level 4,5.

### Questionnaire

The questionnaire was based on the 2009 questionnaire 2, updated in the light of the FCTC Article 14 guidelines, then discussed iteratively and agreed by the survey team. The questionnaire contained 51 items, 21 on treatment and 30 specifically on guidelines. The guidelines questions are shown in Tables 1 and 2.

**Table 1:** Guideline content

Question	% Yes (n)
Are the guidelines for the whole healthcare system and all health professionals and other relevant groups?	72 (38)
Do the guidelines recommend brief advice?	93 (49)
Do the guidelines recommend quitlines?	66 (35)
Do the guidelines recommend intensive specialist support?	93 (49)
Do the guidelines recommend medications?	96 (51)
Do the guidelines include evidence on cost effectiveness?	45 (24)
Do the guidelines reference or refer to the Cochrane Library?	68 (36)
Do the guidelines reference or refer to the guidelines of other countries?	68 (36)
Are the guidelines based on another country's guidelines or other guidelines?	55 (29)
Do the guidelines stress the importance of service providers setting an example by not using tobacco?	57 (30)

Note: all questions answered yes/no; the base was the 53 countries with guidelines; missing data ranged from 2 to 8%.

**Table 2:** Guidelines writing process

Question	% Yes (n)
Is there a strategy to disseminate the guidelines?	57 (30)
Did national professional associations participate in drafting or reviewing them?	70 (37)
Are the guidelines formally endorsed by national professional associations?	68 (36)
Are they formally endorsed or supported by your national government?	70 (37)
Do they clearly describe the writing and review process?	66 (35)
Were they peer-reviewed?	72 (38)
Do they clearly state who funded the guidelines?	76 (40)
Did they receive financial support from government or other public health organisations?	77 (41)
Did they receive financial support from the pharmaceutical industry?	15 (8)
Do they include conflict-of-interest statements for all authors?	40 (21)
Do the names and/or logos of any pharmaceutical companies appear in the guidelines?	11 (6)

Note: all questions answered yes/no; the base was the 53 countries with guidelines; missing data ranged from 2 to 8%.

Descriptive analyses were used to show the distribution of our respondents by WHO region and national income category. All analyses were carried out using IBM SPSS statistical software version 19.

## Results

### Survey response rates

We received responses from 121 (73%) of the 166 countries surveyed.

**Figure 1:**
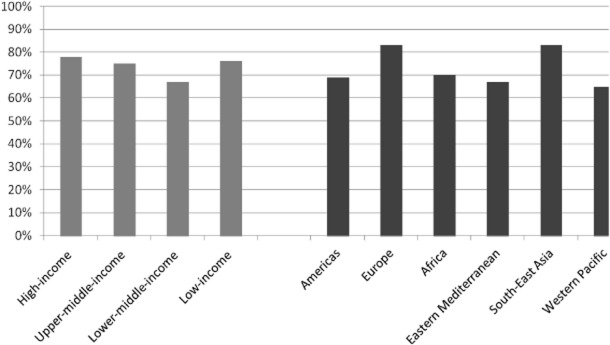
Survey response rates by region and income level

Response rates ranged from 65% of countries surveyed in the Western Pacific region to 83% in Europe and Southeast Asia, and from 67% of surveyed lower-middle-income countries to 78% of surveyed high-income countries (Fig. 1). Our United Arab Emirates contact completed the survey only for Abu Dhabi.

### Countries with guidelines by region and income level

Fifty-three of the 121 countries (44%) reported having treatment guidelines. Twenty-seven (75%) of the high-income countries, 15 (42%) upper-middle-income countries, 9 (30%) lower-middle-income countries and 2 (11%) low-income countries had guidelines.

**Figure 2:**
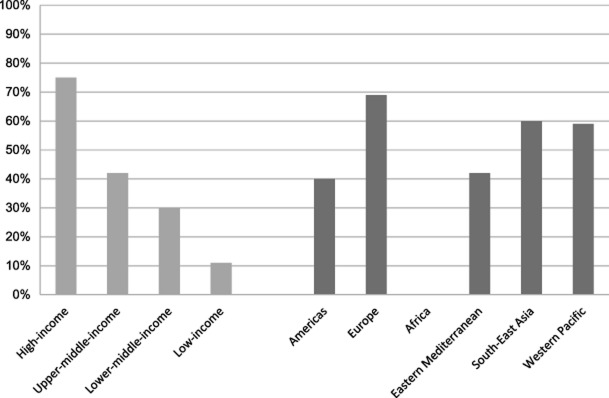
Countries that have treatment guidelines by region and income level

Eight (40%) countries from the Americas, 27 (69%) from the European region, 5 (42%) from the Eastern Mediterranean, 3 (60%) from Southeast Asia and 10 (59%) from the Western Pacific region had guidelines. None from the African region had treatment guidelines (Fig. 2).

### Guideline content

The base for the figures given in the following sections is the 53 countries that have guidelines; key guideline content is shown in Table 1.

Thirty-eight guidelines (72%) were for the whole health-care system and all health professionals. Those not for the whole health-care system were specifically for primary care, nursing, pharmacy, dentists, pregnant tobacco users, doctors outside primary care, teachers, churches, psychiatric patients or psychiatrists.

### Key guideline recommendations

Almost all guidelines recommended brief advice (93%), intensive specialist support (93%) and medications (96%), and 66% recommended quitlines. Of the 51 guidelines that recommended medications (only 1 did not recommend medications), all recommended nicotine replacement therapy, 46 (90%) recommended bupropion, 38 (75%) recommended varenicline, 6 (12%) recommended cytisine and 4 (8%) recommended nortriptyline.

### Other guideline content

Forty-five percent of guidelines included evidence on cost-effectiveness, 68% referenced or referred to the Cochrane library, 68% referenced or referred to guidelines of other countries, and 55% were based on those of at least one other country. The USA, UK and New Zealand were the three countries whose guidelines were most frequently referenced or referred to, or upon which national guidelines were based. Fifty-seven percent stressed the importance of service providers setting an example by not using tobacco.

### Guideline writing process

Fifty-seven percent of guidelines had a dissemination strategy (Table 2). National professional associations were involved in drafting 70%, while 68% were endorsed by national professional associations. Of the guidelines that were formally endorsed by national professional associations, 78% of them were endorsed by 1–9 associations, while 22% were endorsed by 10 or more. Seventy percent of guidelines were formally endorsed or supported by the national government. Sixty-six percent of guidelines clearly described the writing and review process, and 72% were peer reviewed.

Seventy-six percent of guidelines stated who funded them; 77% received financial support from government or other public health organizations; 15% received financial support from the pharmaceutical industry; and 11% contained the names and/or logos of pharmaceutical companies, while 40% included conflict of interest statements for all authors.

Twenty-three guidelines (43%) were published in or after 2010. Several guidelines were published in more than one place. Forty-three (81%) were published as a report or a book, 29 (55%) were published online, 12 (23%) were published in peer reviewed scientific journals and 1 (2%) was published as a supplement to medical journals.

## Discussion

In this global tobacco treatment survey, just under half of the countries surveyed had national treatment guidelines, and our results suggest that most were broadly in line with the evidence base, recommending brief advice, intensive specialist support, medications and quitlines, and most were peer reviewed. Most were developed in a collaborative way, involving national professional associations in drafting and being formally supported by government. Most clearly described the writing and review process; however, fewer than half included conflict of interest statements for all authors, and some received financial support from the pharmaceutical industry and even included the names and logos of pharmaceutical companies.

The main limitation of our study is that there was no way of verifying the accuracy of survey responses. However, we selected our survey contacts as carefully as we could by using tobacco treatment specialists known to us, and other tobacco control specialists recommended by professional colleagues and highly respected organisations. The process of identifying guidelines was limited in that it was not always possible to identify a contact in all Parties to the FCTC and, in cases where we did identify contacts, to get responses from them despite sending reminder emails. Nevertheless, we did get in touch with contacts in all but 10 Parties to the FCTC, and, based on the knowledge we have of guidelines from our previous survey 2 and the guidelines available on http://www.treatobacco.net, we believe that our sample is highly likely to have captured virtually all, if not all, existing guidelines.

To our knowledge this is the largest ever detailed global survey of tobacco dependence treatment, with a response rate of 73% (121 countries). Results reported here are for 68% of FCTC Parties, and show good global representation, including well over half of all World Bank income categories and WHO regions. The lowest response rate by region was in the Western Pacific region (65%), and the highest in Europe and Southeast Asia (83%).

Fifty-three countries (44%) reported having guidelines and 38 (31%) reported that these guidelines were comprehensive, meaning for the whole health-care system and all health-care professionals. There was a linear relationship between income level and having guidelines, with results ranging from 75% of high-income countries to 11% of low-income countries (Fig. 2). Guideline development can be time consuming and expensive, and this may suggest that development of treatment guidelines is not a priority for middle- and low-income countries. None of the Parties surveyed from the African region had guidelines; more than half the countries in the region are low-income and almost a quarter are lower-middle-income 5. Interestingly, Africa has the lowest annual per capita cigarette consumption of all the WHO regions 6 and perhaps this, inter alia, makes treatment guidelines a low priority for Parties in the region. Lack of money and lack of expertise were cited as reasons for not having treatment guidelines in a previous survey 2.

The apparently low priority given to development of treatment guidelines may also reflect a rational response not just to their potential cost but also to the broad content of the FCTC itself 7 and the MPOWER measures 8–10. These emphasise the implementation of policies, such as smoke-free legislation, higher taxation, advertising bans, large graphic health warnings and education on the dangers of tobacco, in order to promote cessation at population level. Logically, until those measures are in place and generating demand for cessation support, it would not make sense to devote substantial resources to developing such support. Furthermore, at the start of our survey it had only been 1 year since the FCTC Article 14 guidelines were adopted, which is very little time in which to develop guidelines in accordance with FCTC recommendations. It is therefore likely that some Parties are currently developing guidelines. Given that 57% of guidelines in our survey were published before 2010, arguably even Parties with guidelines may consider updating them at some point in the near future.

We already commented that most guidelines recommended brief advice, intensive specialist support, medications and quitlines. The Cochrane library was referenced or referred to by the majority of guidelines, suggesting that the FCTC recommendation that guidelines should be ‘based on scientific evidence’ is being taken seriously. Over half of the guidelines were based on those of other countries, mostly the US and UK guidelines, possibly a very practical cost-saving approach.

A key finding, however, was that only 57% of countries said they had a strategy to disseminate their guidelines. Given that, logically, national guidelines will form the basis for treatment services, the lack of a dissemination plan is of concern, and the FCTC Article 14 guidelines clearly state that national guidelines should include a dissemination plan. However, in some countries the existence of official national guidelines is a powerful statement of the importance of the issue, so even without a dissemination strategy, guidelines can be very important.

Rates of tobacco use by health professionals are very high in some countries 11, which is why the FCTC Article 14 guidelines recommend that guidelines stress the importance of health-care providers not using tobacco and of helping them stop. In spite of this, only 57% of guidelines stress this.

The high proportions of guidelines that involved national professional associations in their drafting (70%), which were formally supported by government (70%) and by public health organisations (68%), and which received financial support from government or other public health organisations (77%), suggests that the majority of the guidelines were produced as a result of genuine collaboration between relevant stakeholders, a recommendation of the FCTC Article 14 guidelines. We believe such collaboration is important for at least two reasons: no country has unlimited resources so all resources available should be used; and the failure of genuine collaboration can result in real confusion, as in one country we surveyed that has fractured national consensus on treatment and three ‘competing’ guidelines. Furthermore, it is likely that this collaboration resulted in the high levels of endorsement from national health professional organisations and governments.

Fifteen percent of guidelines in our survey received financial support from the pharmaceutical industry, 11% carried pharmaceutical company names and/or logos (in one case a logo on the title page), and fewer than half included conflict-of-interest statements for all authors. The FCTC Article 14 guidelines do not actually prohibit pharmaceutical company support, but do state that the development process must be protected from vested interests. Arguably it is at least unwise to develop guidelines with such industry support, but, in our opinion, it is entirely unacceptable to promote company names and logos in guidelines. If pharmaceutical company funding is considered necessary, then there must be very clear rules to guarantee the independence and integrity of the guidelines. However guideline development need not be expensive, as tools are being developed to assist countries, which would greatly reduce costs and which will be available in 2013. We believe this route to be far preferable to pharmaceutical company funding, which will, inevitably, in many peoples' views undermine guidelines. Finally, we believe that full declaration of all interests is essential.

Overall, our findings suggest that the development of guidelines is not yet a priority for many Parties to the FCTC, but that most existing guidelines followed Article 14 guideline recommendations quite closely. The aim of treatment guidelines is to assist health care practitioners and patients in making decisions regarding tobacco dependence and cessation [1], and yet the majority of FCTC Parties do not have guidelines. The cost of producing guidelines appears to be a significant barrier; therefore, initiatives to provide technical and/or financial support to resource limited countries are likely to expedite guidelines development in these Parties.

The finding that the majority of existing guidelines are based on the guidelines of other countries shows that guideline production need not necessarily start from scratch and may provide a way to help resource limited countries. The development and dissemination of a guidelines ‘template’, so to speak, which FCTC Parties could subsequently tailor to suit their respective national situations may translate into significant time and cost savings for Parties and spur progress with guidelines development. Although the focus of this report is the FCTC Article 14 significant gains can only be made if other complementary FCTC Articles are implemented concurrently by Parties.

## Supporting information

**Appendix S1** Tobacco Treatment Survey 2011/12.
