# Supplementary material for: A survey of tobacco dependence treatment guidelines in 121 countries
Source: Addiction. 2013 Apr 22;108(8):1470–5. doi: 10.1111/add.12158 (PMC3759700; doi:10.1111/add.12158)
Supplement: Supplementary file 1 [file add0108-1470-SD1.docx]

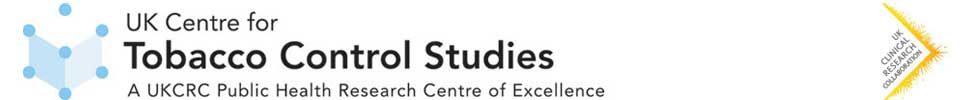


| **Encuesta sobre el tratamiento anti tabáquico 2011/12** |
| --- |

| Agradecemos su disposición para participar de esta encuesta que forma parte del informe paralelo del programa de la Alianza del Convenio Marco (Framework Convention Alliance o FCA como son sus siglas en inglés).  Completar el cuestionario le ocupará aproximadamente entre 10 y 15 minutos de su tiempo.  Desde ya muchas gracias.  Martin Raw ( martin@martinraw.com ) y Hemba Pine-Abata ( mcxhp@nottingham.ac.uk ) en nombre del equipo de investigación de las Universidades de Nottingham y Harvard |
| --- |

| **Datos personales** |
| --- |

| ***1) Sírvase aportar la mayor información posible** |
| --- |
| Nombre: |
| Institución/Organización: |
| País: |
| Dirección de correo electrónico: |

| **2) Puesto** |
| --- |
| Puesto: |

| **3) Nombre de Skype** |
| --- |
| Nombre de Skype: |

| **Cuestionario sobre el tratamiento de la dependencia del tabaco en su país** |
| --- |

| ***4) ¿Hay alguna persona identificada oficialmente en el gobierno (o contratada por el gobierno) que sea responsable de los tratamientos de la dependencia del tabaco?** | |
| --- | --- |
| Si |  |
| No |  |

| ***5) En su país ¿se realizan campañas en los medios masivos de comunicación promocionando la cesación tabáquica?** | |
| --- | --- |
| Si |  |
| No |  |

| ***6) En su país ¿existen líneas telefónicas de ayuda para dejar de fumar (quitline)?** | |
| --- | --- |
| Si, hay una línea nacional o líneas en las regiones más importantes del país |  |
| No |  |

| **Si la respuesta es no, continúe con la pregunta número 9** |
| --- |

| **Si la respuesta es afirmativa** |
| --- |

| ***7) ¿Son líneas gratuitas?** | |
| --- | --- |
| Si |  |
| No |  |

| **8) La línea de ayuda (quitline):** | | | |
| --- | --- | --- | --- |
|  | Si | No | No sabe |
| Existen personas que contestan siempre o casi siempre |  |  |  |
| Ofrece varias sesiones terapéuticas con consejeros que devuelven las llamadas brindando apoyo permanente |  |  |  |
| Los deriva a especialistas locales para los tratamientos |  |  |  |
| Brinda información acerca de los medicamentos para dejar de fumar |  |  |  |
| Ofrece medicamentos para dejar de fumar a los que llaman |  |  |  |

| ***9) En su país ¿existen instituciones especializadas (expertos, unidades o centros) para el tratamiento de la dependencia del tabaco que brinden apoyo individual o grupal impartido por profesionales entrenados?** | |
| --- | --- |
| Si, una red de tratamiento de apoyo que abarca todo el país |  |
| Si, tratamiento de apoyo pero solo en ciertas áreas |  |
| No |  |

| ***10) ¿Pueden los fumadores obtener ayuda para dejar de fumar de los siguientes entornos?** | | | |
| --- | --- | --- | --- |
|  | Si con facilidad / de todos o de la mayoría | Difícilmente / solo de algunos | No / solo de unos pocos o ninguno |
| Medicina general/ medicina familiar |  |  |  |
| Farmacéuticos |  |  |  |
| Dentistas |  |  |  |
| Hospitales |  |  |  |
| Centros de adicciones |  |  |  |
| Lugares de trabajo |  |  |  |
| Instituciones educativas |  |  |  |
| Prisiones |  |  |  |
| Curadores/Sanadores |  |  |  |
| En internet |  |  |  |

| **11) Los siguientes medicamentos ¿están disponibles en su país? Si es así, ¿cómo se comercializan?** | | | |
| --- | --- | --- | --- |
| POR FAVOR MARQUE TODOS LOS QUE CORRESPONDA | | | |
| Venta libre: se pueden comprar en supermercados o comercios de barrio. Farmacia: se puede adquirir sin receta solo por medio de un farmacéutico. Receta: sólo pueden comprarse con receta médica. | | | |
|  | Venta Libre | Farmacias | Con Receta |
| Chicles de nicotina |  |  |  |
| Parches de nicotina |  |  |  |
| Comprimidos sublinguales de nicotina |  |  |  |
| Comprimidos masticables de nicotina |  |  |  |
| Inhaladores de nicotina |  |  |  |
| Espray nasal de nicotina |  |  |  |
| Bupropión |  |  |  |
| Vareniclina |  |  |  |
| Cistisina |  |  |  |
| Clonidina |  |  |  |
| Nortriptilina |  |  |  |

| **12) ¿Los siguientes medicamentos son accesibles en costo para la mayoría de los fumadores en su país?** | | |
| --- | --- | --- |
|  | Si | No |
| Chicles de nicotina |  |  |
| Parches de nicotina |  |  |
| Comprimidos sublinguales de nicotina |  |  |
| Comprimidos masticables de nicotina |  |  |
| Inhaladores de nicotina |  |  |
| Espray nasal de nicotina |  |  |
| Bupropión |  |  |
| Vareniclina |  |  |
| Cistisina |  |  |
| Clonidina |  |  |
| Nortriptilina |  |  |

| **Cuestionario sobre los lineamientos del tratamiento en su país** |
| --- |

| ***13) ¿Existen en su país guías nacionales para el tratamiento de la dependencia del tabaco?** | |
| --- | --- |
| Si |  |
| No |  |

| **Si la respuesta es no, continúe con la pregunta número 34** |
| --- |

| **Si la respuesta es afirmativa** |
| --- |

| ***14) ¿En qué año se publicó la última versión de las guías?** |
| --- |
|  |

| **15) ¿En qué año se publicó la versión o versiones anteriores de las guías?** |
| --- |
|  |

| **16) ¿Dónde fueron publicadas las guías?** | |
| --- | --- |
| En una revista científica arbitrada por pares |  |
| Como un informe/libro |  |
| Online |  |
| Otras (especificar): | |

| **17) ¿Existe alguna estrategia o plan para difundir las guías?** | |
| --- | --- |
| Si |  |
| No |  |

| **18) Elaboración de las Guías** | | |
| --- | --- | --- |
|  | Si | No |
| ¿Describen claramente el proceso de redacción y revisión? |  |  |
| ¿Establece claramente quién financió las guías? |  |  |
| ¿Incluye declaraciones de conflicto de interés de todos los autores? |  |  |
| ¿Recibieron respaldo financiero de la industria farmacológica? |  |  |
| ¿Recibieron respaldo financiero del gobierno u otras organizaciones de salud pública? |  |  |
| ¿Aparecen en las guías el nombre o logotipo de alguna empresa farmacológica? |  |  |

| **19) Características principales de las guías** | | |
| --- | --- | --- |
|  | Si | No |
| ¿Son las guías para todo el sistema sanitario, todos los profesionales de la salud y otros grupos relevantes? |  |  |

| **20) Si la respuesta es no, especificar a que grupo o grupos de profesionales, establecimientos o clientes están dirigidas** | |
| --- | --- |
| Centros sanitarios |  |
| Enfermería |  |
| Farmacia |  |
| Dentistas |  |
| Embarazadas que fuman |  |
| Otros (especificar): | |

| **21) ¿Qué recomiendan las guías?** | | |
| --- | --- | --- |
|  | Si | No |
| ¿Consejo breve? |  |  |
| ¿Líneas de ayuda (quitlines)? |  |  |
| ¿Apoyo intensivo de especialistas? |  |  |

| **22) ¿Las guías recomiendan medicamentos?** | |
| --- | --- |
| Si |  |
| No |  |

| **23) ¿Que tipo de medicamentos recomiendan las guías?** | |
| --- | --- |
| Nicotina |  |
| Bupropión |  |
| Vareniclina |  |
| Cistisina |  |
| Otros (especificar): | |

| **24) ¿Las guías remarcan la importancia que los proveedores de servicios den el ejemplo de no fumar?** | |
| --- | --- |
| Si |  |
| No |  |

| **25) ¿Están las guías avaladas formalmente por asociaciones nacionales de profesionales?** | |
| --- | --- |
| Si |  |
| No |  |

| **26) Si la respuesta es afirmativa, ¿aproximadamente cuantas?** | |
| --- | --- |
| 1 a 9 |  |
| 10 o más |  |

| **27) Redacción de las guías** | | |
| --- | --- | --- |
|  | Si | No |
| ¿Están arbitradas por pares? |  |  |
| ¿Están aprobadas o respaldadas formalmente por el gobierno nacional? |  |  |
| ¿Participaron las asociaciones profesionales en la redacción o revisión de las mismas? |  |  |

| **28) ¿Las guías incluyen datos sobre la relación costo-efectividad?** | |
| --- | --- |
| Si |  |
| No |  |

| **29) ¿Las guías refieren o hacen referencia a la Biblioteca Cochrane?** | |
| --- | --- |
| Si |  |
| No |  |

| **30) ¿Las guías refieren o hacen referencia a guías de otros países?** | |
| --- | --- |
| Si |  |
| No |  |

| **31) Si la respuesta es afirmativa, ¿a qué país o países?** |
| --- |
|  |

| **32) ¿Las guías se fundamentan o basan en guías de otros países o en otras guías?** | |
| --- | --- |
| Si |  |
| No |  |

| **33) Si la respuesta es afirmativa, ¿en qué país o países?** |
| --- |
|  |

| **Finalmente** |
| --- |

| **34) ¿Tiene su país un plan nacional oficial para promover la cesación tabáquica y proporcionar tratamiento a la dependencia del tabaco?** | |
| --- | --- |
| Si |  |
| No |  |

| **35)Su país:** | | | |
| --- | --- | --- | --- |
|  | Si | No | No sabe |
| ¿Tiene un presupuesto claramente identificado para el tratamiento? |  |  |  |
| En la historia clínica ¿tiene un registro obligatorio de la condición de consumo de tabaco del paciente? |  |  |  |
| ¿Promueve/fomenta el consejo breve en los centros de servicios existentes de (pero no limitado solo a ellos) la tuberculosis, HIV/AID, etc.? |  |  |  |
| ¿Ofrece ayuda para dejar de fumar a los agentes de salud y a otros grupos relevantes? |  |  |  |
| ¿Tiene una estrategia de financiación nacional para investigación en la cesación tabáquica? |  |  |  |
| ¿Monitorea el uso de los servicios de tratamiento (incluyendo su rendimiento y calidad)? |  |  |  |
| ¿Tiene estándares nacionales de capacitación? |  |  |  |

| **36) Sírvase añadir aquí cualquier otro comentario que le gustaría hacer con las referencias pertinentes si las hubiera.** |
| --- |
|  |

| **Encuesta completa** |
| --- |

Muchas gracias.

Martin Raw ( martin@martinraw.com ) y Hemba Pine-Abata ( mcxhp@nottingham.ac.uk )
