# Supplementary material for: A survey of tobacco dependence treatment guidelines in 121 countries
Source: Addiction. 2013 Apr 22;108(8):1470–5. doi: 10.1111/add.12158 (PMC3759700; doi:10.1111/add.12158)
Supplement: Supplementary file 2 [file add0108-1470-SD2.doc]

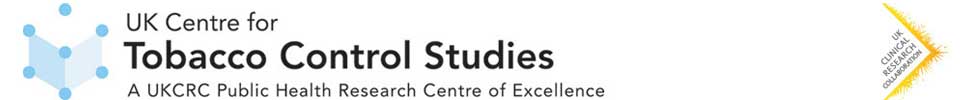


| **Enquête Traitement Tabac 2011/12** |
| --- |

| Merci d'avoir accepté de prendre part à cette enquête faisant partie du programme de l'Alliance sur la Convention-cadre (FCA) de surveillance de la performance des parties.  Remplir ce questionnaire devrait vous prendre 10 à 15 minutes.  Merci beaucoup.  Martin Raw ( martin@martinraw.com ) et Hemba Pine-Abata ( mcxhp@nottingham.ac.uk ) de la part de l'équipe responsable de l'enquête aux universités de Nottingham et de Harvard |
| --- |

| **Vos coordonnées** |
| --- |

| ***1) Merci de fournir le plus de précisions possibles** |
| --- |
| Nom: |
| Institution/Organisation: |
| Pays: |
| Courriel: |

| **2) Votre position** |
| --- |
| Position: |

| **3) Votre adresse skype** |
| --- |
| Skype name: |

| **Questions concernant le traitement de la dépendance au tabac dans votre pays** |
| --- |

| ***4) Y a-t-il une personne au gouvernement (ou sous contrat du gouvernement) officiellement en charge des questions sur le traitement de la dépendance au tabac?** | |
| --- | --- |
| Oui |  |
| Non |  |

| ***5) Votre pays fait-il des campagnes de masse pour promouvoir l'arrêt du tabac?** | |
| --- | --- |
| Oui |  |
| Non |  |

| ***6) Votre pays possède-t-il une ligne d'aide à l'arrêt téléphonique?** | |
| --- | --- |
| Oui, une ligne d'aide à l'arrêt nationale ou des lignes d'aide à l'arrêt dans les principales régions |  |
| Non |  |

| **Si non, allez directement à la question 9** |
| --- |

| **Si oui** |
| --- |

| ***7) Est-ce une ligne gratuite?** | |
| --- | --- |
| Oui |  |
| Non |  |

| **8) Est-ce qu'elle:** | | | |
| --- | --- | --- | --- |
|  | Oui | Non | Ne sais pas |
| Est ouverte tout le temps ou presque tout le temps |  |  |  |
| Offre des séances multiples avec des tabacologues qui rappellent et offrent un suivi |  |  |  |
| Renvoie le cas échéant vers un tabacologue local |  |  |  |
| Propose des informations sur les médicaments d'aide à l'arrêt |  |  |  |
| Offre des traitements médicamenteux aux appelants |  |  |  |

| ***9) Votre pays a-t-il des centres spécialisés dans l'aide à l'arrêt du tabac (experts ou unités/cliniques) proposant une aide individuelle ou en groupe réalisée par des professionnels formés?** | |
| --- | --- |
| Oui, un réseau d'aide à l'arrêt dans l'ensemble du pays |  |
| Oui, un réseau d'aide à l'arrêt, mais seulement dans certaines régions |  |
| Non |  |

| ***10) Les fumeurs peuvent-ils se faire aider dans les lieux suivants?** | | | |
| --- | --- | --- | --- |
|  | Oui, facilement / partout ou presque | Pas très facilement / pas partout | Pas vraiment / rares endroits ou pas du tout |
| Généralistes |  |  |  |
| Pharmaciens |  |  |  |
| Dentistes |  |  |  |
| Hôpitaux |  |  |  |
| Centres d'Addiction |  |  |  |
| Au travail |  |  |  |
| Dans les lieux d'enseignement (écoles...) |  |  |  |
| Dans les prisons |  |  |  |
| Médecines traditionnelles |  |  |  |
| Par internet |  |  |  |

| **11) Ces médicaments sont-ils disponibles dans votre pays, et si oui, comment sont-ils mis sur le marché?** | | | |
| --- | --- | --- | --- |
| MERCI DE COCHER TOUTES LES CASES APPROPRIEES | | | |
| Magasins: peut être acheté au supermarché ou dans une épicerie de quartier; Pharmacies: ne peut être acheté sans ordonnance qu'en pharmacie; Prescription: ne peut être obtenu qu'avec une ordonnance médicale. | | | |
|  | Magasins | Pharmacies | Prescription |
| TNS gomme |  |  |  |
| TNS patch |  |  |  |
| TNS comprimé sublingual |  |  |  |
| TNS comprimé à sucer |  |  |  |
| TNS inhaleur |  |  |  |
| TNS spray nasal |  |  |  |
| Bupropion |  |  |  |
| Varénicline |  |  |  |
| Cytisine |  |  |  |
| Clonidine |  |  |  |
| Nortriptyline |  |  |  |

| **12) Ces médicaments sont-ils accessibles (bon marché) pour la plupart des fumeurs de votre pays?** | | |
| --- | --- | --- |
|  | Oui | Non |
| TNS gomme |  |  |
| TNS patch |  |  |
| TNS comprimé sublingual |  |  |
| TNS comprimé à sucer |  |  |
| TNS inhaleur |  |  |
| TNS spray nasal |  |  |
| Bupropion |  |  |
| Varénicline |  |  |
| Cytisine |  |  |
| Clonidine |  |  |
| Nortriptyline |  |  |

| **Questions sur les recommandations de bonnes pratiques cliniques dans votre pays** |
| --- |

| ***13) Votre pays a-t-il des recommandations de bonnes pratiques cliniques nationales pour l'aide à l'arrêt du tabac?** | |
| --- | --- |
| Oui |  |
| Non |  |

| **Si non, allez directement à la question 34** |
| --- |

| **Si oui** |
| --- |

| ***14) En quelle année ont été publiées les recommandations les plus récentes?** |
| --- |
|  |

| **15) En quelle(s) année(s) a/ont été publiée(s) la/les précédente(s) version(s)?** |
| --- |
|  |

| **16) Où sont publiées ces recommandations?** | |
| --- | --- |
| Dans un journal scientifique à comité de lecture |  |
| Sous forme d'un livre/rapport |  |
| Sur internet |  |
| Ailleurs (merci de préciser): | |

| **17) Existe-t-il une stratégie de diffusion de ces recommandations?** | |
| --- | --- |
| Oui |  |
| Non |  |

| **18) La procédure utilisée pour ces recommandations** | | |
| --- | --- | --- |
|  | Oui | Non |
| Décrivent-elles précisément la procédure d'écriture et de relecture? |  |  |
| Décrivent-elles précisément qui a financé ces recommandations? |  |  |
| Présentent-elles des déclarations de conflits d'intérêts de tous les auteurs? |  |  |
| Ont-elles reçu un soutien financier de l'industrie pharmaceutique? |  |  |
| Ont-elles reçu un soutien financier du gouvernement ou d'autres organisations de santé publique? |  |  |
| Les noms et/ou les logos de compagnies pharmaceutiques apparaissent-ils dans ces recommandations? |  |  |

| **19) Quelques caractéristiques clés de ces recommandations** | | |
| --- | --- | --- |
|  | Oui | Non |
| Ces recommandations sont-elles destinées à l'ensemble du système de santé et à tous les professionnels de santé, et autres groupes concernés? |  |  |

| **20) Si non, merci de spécifier pour qui sont-elles (profession, cadre/milieu, groupe...)** | |
| --- | --- |
| Médecins généralistes |  |
| Infirmières |  |
| Pharmacies |  |
| Dentistes |  |
| Femmes enceintes |  |
| Autres (précisez): | |

| **21) Ces recommandations préconisent-elles** | | |
| --- | --- | --- |
|  | Oui | Non |
| Le conseil minimal? |  |  |
| Les lignes téléphoniques d'arrêt? |  |  |
| L'aide intensive d'un spécialiste? |  |  |

| **22) Ces recommandations préconisent-elles des médications?** | |
| --- | --- |
| Oui |  |
| Non |  |

| **23) Quels sont les médicaments recommandés?** | |
| --- | --- |
| Substituts nicotiniques |  |
| Buproprion |  |
| Varénicline |  |
| Cytisine |  |
| Autres (précisez): | |

| **24) Les recommandations insistent-elles sur l'importance pour un tabacologue de ne pas fumer, afin de montrer l'exemple?** | |
| --- | --- |
| Oui |  |
| Non |  |

| **25) Les recommandations sont-elles formellement approuvées par des associations professionnelles nationales?** | |
| --- | --- |
| Oui |  |
| Non |  |

| **26) Si oui, combien approximativement?** | |
| --- | --- |
| 1 à 9 |  |
| 10 ou plus |  |

| **27) Le processus d'écriture** | | |
| --- | --- | --- |
|  | Oui | Non |
| Ont-elles été relues par un comité de lecture? |  |  |
| Sont-elles formellement approuvées ou supportées par votre gouvernement? |  |  |
| Des associations professionnelles nationales ont-elles participé à l'écriture ou à la relecture? |  |  |

| **28) Les recommandations incluent-elles des preuves de coût-efficacité?** | |
| --- | --- |
| Oui |  |
| Non |  |

| **29) Ces recommandations font-elles référence aux revues de la Cochrane Library?** | |
| --- | --- |
| Oui |  |
| Non |  |

| **30) Ces recommandations font-elles référence à des recommandations d'autres pays?** | |
| --- | --- |
| Oui |  |
| Non |  |

| **31) Si oui, quel(s) pays?** |
| --- |
|  |

| **32) Ces recommandations sont-elles basées sur les recommandations d'un autre pays ou sur d'autres recommandations?** | |
| --- | --- |
| Oui |  |
| Non |  |

| **33) Si oui, quel(s) pays?** |
| --- |
|  |

| **Finalement** |
| --- |

| **34) Votre pays a-t-il une stratégie nationale officielle pour promouvoir l'arrêt du tabac et proposer le traitement de la dépendance au tabac?** | |
| --- | --- |
| Oui |  |
| Non |  |

| **35) Est-ce que votre pays:** | | | |
| --- | --- | --- | --- |
|  | Oui | Non | Ne sais pas |
| a un budget clairement identifié pour les traitements? |  |  |  |
| possède un système obligatoire afin d'enregistrer le tabagisme des patients dans le dossier médical? |  |  |  |
| encourage/promeut le conseil minimal dans les services comme ceux prenant en charge (mais pas seulement ceux-ci) la tuberculose, le VIH/SIDA, etc...? |  |  |  |
| offre aux soignants et à d'autres groupes la possibilité de les aider à arrêter de fumer? |  |  |  |
| possède une stratégie de recherche nationale financée sur l'arrêt du tabac? |  |  |  |
| contrôle l'utilisation de l'aide à l'arrêt du tabac (incluant l'efficacité et la qualité)? |  |  |  |
| propose une formation à l'aide à l'arrêt du tabac standardisée? |  |  |  |

| **36) Merci d'indiquer ici les commentaires ou remarques que vous souhaitez faire, incluant toute(s) référence(s) que vous jugeriez utile(s)** |
| --- |
|  |

| **L'enquête est terminée** |
| --- |

Merci beaucoup de votre collaboration.

Martin Raw ( martin@martinraw.com ) et Hemba Pine-Abata ( mcxhp@nottingham.ac.uk )
