# Supplementary material for: A survey of tobacco dependence treatment guidelines in 121 countries
Source: Addiction. 2013 Apr 22;108(8):1470–5. doi: 10.1111/add.12158 (PMC3759700; doi:10.1111/add.12158)
Supplement: Supplementary file 3 [file add0108-1470-SD3.docx]

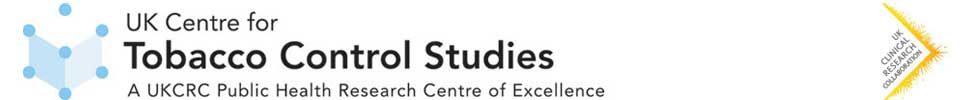


| **Tobacco Treatment Survey 2011/12** |
| --- |

| Thank you for agreeing to take part in this survey, which is part of the shadow reporting programme of the Framework Convention Alliance (FCA).  The questionnaire should take approximately 10 to 15 minutes to complete.  Thank you very much.  Martin Raw ( [martin@martinraw.com](mailto:martin@martinraw.com) ) and Hemba Pine-Abata ( [mcxhp@nottingham.ac.uk](mailto:mcxhp@nottingham.ac.uk) ) on behalf of the survey team at Nottingham and Harvard Universities |
| --- |

| **Contact information** |
| --- |

| ***1) Please supply as much information as you can** |
| --- |
| Name: |
| Institution/Organisation: |
| Country: |
| Email address: |

| **2) Your position** |
| --- |
| Position: |

| **3) Your skype name** |
| --- |
| Skype name: |

| **Questions about tobacco dependence treatment in your country** |
| --- |

| ***4) Is there an officially identified person in government (or contracted by government) who is responsible for tobacco dependence treatment?** | |
| --- | --- |
| Yes |  |
| No |  |

| ***5) Does your country run mass media campaigns promoting cessation?** | |
| --- | --- |
| Yes |  |
| No |  |

| ***6) Does your country have a telephone quitline?** | |
| --- | --- |
| Yes, a national quitline or quitlines in all major regions of the country |  |
| No |  |

| **If no go to question 9** |
| --- |

| **If yes** |
| --- |

| ***7) Is it free to callers calling in?** | |
| --- | --- |
| Yes |  |
| No |  |

| **8) Does the quitline:** | | | |
| --- | --- | --- | --- |
|  | Yes | No | Don't know |
| Have people answering always or almost always |  |  |  |
| Offer multiple sessions with counsellors calling back offering ongoing support |  |  |  |
| Refer to local specialist treatment services |  |  |  |
| Offer information about tobacco cessation medications |  |  |  |
| Offer tobacco cessation medications to callers |  |  |  |

| ***9) Does your country have specialised tobacco dependence treatment facilities (experts or units/clinics) offering individual or group support delivered by trained professionals?** | |
| --- | --- |
| Yes a network of treatment support covering the whole country |  |
| Yes treatment support but only in selected areas |  |
| No |  |

| ***10) Can tobacco users get help to stop in the following settings?** | | | |
| --- | --- | --- | --- |
|  | Yes easily / from all or most | Not easily / from some | No / from very few or none |
| General/family practice |  |  |  |
| Pharmacists |  |  |  |
| Dentists |  |  |  |
| Hospitals |  |  |  |
| Addiction services |  |  |  |
| Work places |  |  |  |
| Educational institutions |  |  |  |
| Prisons |  |  |  |
| Traditional health practitioners / healers |  |  |  |
| From the internet |  |  |  |

| **11) Are the following medications available in your country and if so how are they licensed?** | | | |
| --- | --- | --- | --- |
| PLEASE TICK ALL THAT APPLY | | | |
| General sale: can be purchased from supermarkets or corner shops for example; Pharmacy: can only be purchased over-the-counter from a pharmacist; Prescription: only obtainable with a doctor's prescription. | | | |
|  | General sale | Pharmacy | Prescription |
| NRT gum |  |  |  |
| NRT patch |  |  |  |
| NRT sublingual tablet |  |  |  |
| NRT lozenge |  |  |  |
| NRT inhaler |  |  |  |
| NRT nasal spray |  |  |  |
| Bupropion |  |  |  |
| Varenicline |  |  |  |
| Cytisine |  |  |  |
| Clonidine |  |  |  |
| Nortriptyline |  |  |  |

| **12) Are the following medications easily affordable to most tobacco users in your country?** | | |
| --- | --- | --- |
|  | Yes | No |
| NRT gum |  |  |
| NRT patch |  |  |
| NRT sublingual tablet |  |  |
| NRT lozenge |  |  |
| NRT inhaler |  |  |
| NRT nasal spray |  |  |
| Bupropion |  |  |
| Varenicline |  |  |
| Cytisine |  |  |
| Clonidine |  |  |
| Nortriptyline |  |  |

| **Questions about treatment guidelines in your country** |
| --- |

| ***13) Does your country have national guidelines for the treatment of tobacco dependence?** | |
| --- | --- |
| Yes |  |
| No |  |

| **If no go to question 34** |
| --- |

| **If yes** |
| --- |

| ***14) What year was the most recent version of the guidelines published?** |
| --- |
|  |

| **15) What year was the previous version(s) of the guidelines published?** |
| --- |
|  |

| **16) Where are the guidelines published?** | |
| --- | --- |
| In a peer reviewed scientific journal |  |
| As a report/book |  |
| Online |  |
| Other (please specify): | |

| **17) Is there a strategy to disseminate the guidelines?** | |
| --- | --- |
| Yes |  |
| No |  |

| **18) The guidelines process** | | |
| --- | --- | --- |
|  | Yes | No |
| Do they clearly describe the writing and review process? |  |  |
| Do they clearly state who funded the guidelines? |  |  |
| Do they include conflict-of-interest statements for all authors? |  |  |
| Did they receive financial support from the pharmaceutical industry? |  |  |
| Did they receive financial support from government or other public health organisations? |  |  |
| Do the names and/or logos of any pharmaceutical companies appear in the guidelines? |  |  |

| **19) Some key guideline characteristics** | | |
| --- | --- | --- |
|  | Yes | No |
| Are the guidelines for the whole healthcare system and all health professionals and other relevant groups? |  |  |

| **20) If no please specify which profession, setting or client group/s they are for** | |
| --- | --- |
| Primary care |  |
| Nursing |  |
| Pharmacy |  |
| Dentists |  |
| Pregnant tobacco users |  |
| Other (please specify): | |

| **21) Do the guidelines recommend** | | |
| --- | --- | --- |
|  | Yes | No |
| Brief advice? |  |  |
| Quitlines? |  |  |
| Intensive specialist support? |  |  |

| **22) Do the guidelines recommend medications** | |
| --- | --- |
| Yes |  |
| No |  |

| **23) Which medications do the guidelines recommend?** | |
| --- | --- |
| NRT |  |
| Buproprion |  |
| Varenicline |  |
| Cytisine |  |
| Other (please specify): | |

| **24) Do the guidelines stress the importance of service providers setting an example by not using tobacco?** | |
| --- | --- |
| Yes |  |
| No |  |

| **25) Are the guidelines formally endorsed by national professional associations?** | |
| --- | --- |
| Yes |  |
| No |  |

| **26) If so approximately how many?** | |
| --- | --- |
| 1 to 9 |  |
| 10 or more |  |

| **27) The guidelines writing process** | | |
| --- | --- | --- |
|  | Yes | No |
| Were they peer reviewed? |  |  |
| Are they formally endorsed or supported by your national government? |  |  |
| Did national professional associations participate in drafting or reviewing them? |  |  |

| **28) Do the guidelines include evidence on cost effectiveness?** | |
| --- | --- |
| Yes |  |
| No |  |

| **29) Do the guidelines reference or refer to the Cochrane Library?** | |
| --- | --- |
| Yes |  |
| No |  |

| **30) Do the guidelines reference or refer to the guidelines of other countries?** | |
| --- | --- |
| Yes |  |
| No |  |

| **31) If yes which country/countries?** |
| --- |
|  |

| **32) Are the guidelines based on another country's guidelines or other guidelines?** | |
| --- | --- |
| Yes |  |
| No |  |

| **33) If yes which country/countries?** |
| --- |
|  |

| **Finally** |
| --- |

| **34) Does your country have an official national strategy to promote tobacco cessation and provide tobacco dependence treatment?** | |
| --- | --- |
| Yes |  |
| No |  |

| **35) Does your country:** | | | |
| --- | --- | --- | --- |
|  | Yes | No | Don't know |
| Have a clearly identified budget for treatment |  |  |  |
| Have mandatory recording of patients’ tobacco use status in medical notes |  |  |  |
| Promote/encourage brief advice in existing services like (but not limited to) tuberculosis, HIV/AIDs, etc |  |  |  |
| Offer help to healthcare and other relevant groups stop using tobacco |  |  |  |
| Have a funded national research strategy for tobacco use cessation |  |  |  |
| Monitor the use of treatment services (including throughput and quality) |  |  |  |
| Have national training standards |  |  |  |

| **36) Please add here any further comments you would like to make, including any relevant references if any** |
| --- |
|  |

| **Survey complete** |
| --- |

Thank you very much indeed.

Martin Raw ( [martin@martinraw.com](mailto:martin@martinraw.com) ) and Hemba Pine-Abata ( [mcxhp@nottingham.ac.uk](mailto:mcxhp@nottingham.ac.uk) )
